# 2754. Delafloxacin and Comparator Fluoroquinolone *In Vitro* Resistance Trends in Isolates from Skin and Skin Structure Infections in the USA (2017–2022)

**DOI:** 10.1093/ofid/ofad500.2365

**Published:** 2023-11-27

**Authors:** Cecilia G Carvalhaes, Dee Shortridge, Leonard R Duncan, Mike Huband, Mariana Castanheira

**Affiliations:** JMI Laboratories, North Liberty, IA; JMI Laboratories, North Liberty, IA; JMI Laboratories, North Liberty, IA; JMI Laboratories, North Liberty, IA; JMI Laboratories, North Liberty, IA

## Abstract

**Background:**

Delafloxacin (DLX) is a broad-spectrum fluoroquinolone antibacterial approved in the USA in 2017 for treatment of acute bacterial skin and skin structure infection (ABSSSI). DLX is indicated to treat ABSSSI caused by multiple pathogens, including *Staphylococcus aureus* (methicillin-resistant [MRSA] and methicillin-susceptible [MSSA]), *Streptococcus pyogenes* (SP), *Enterococcus faecalis* (EF), and Gram-negative organisms, including *Escherichia coli* (EC) and *Pseudomonas aeruginosa* (PSA). In this study, the *in vitro* susceptibilities of DLX and comparator fluoroquinolones (FQ), levofloxacin (LEV), moxifloxacin (MOX), and ciprofloxacin (CIP) were determined for US clinical isolates.

**Figure:**
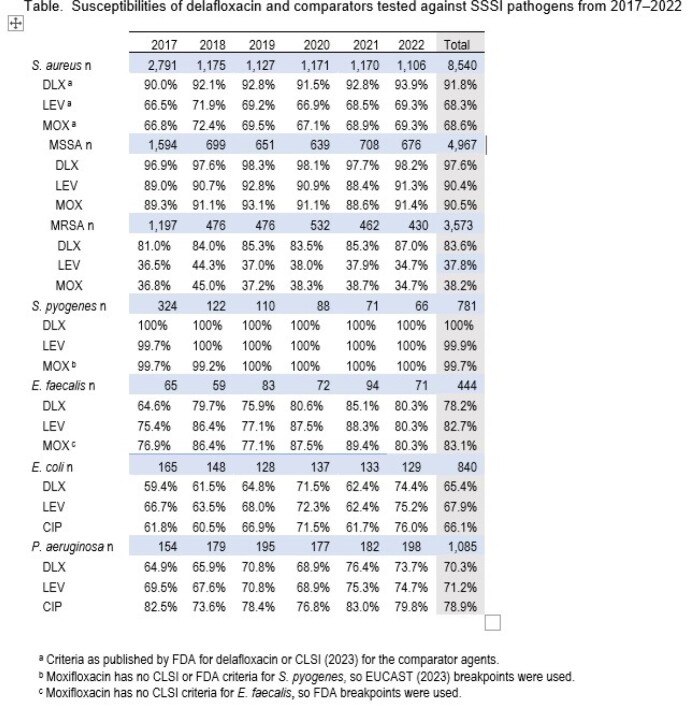

**Methods:**

Isolates from SSSI were consecutively collected at 77 US medical centers participating in the SENTRY Surveillance Program (2017–2022). Sites submitted 1 isolate per patient per infection episode. Isolate identification was determined at each site and confirmed using MALDI-TOF MS at JMI Laboratories. Susceptibility testing was performed according to CLSI broth microdilution methodology. FDA interpretative criteria were used for DLX; CLSI (2023) criteria were applied to comparators. MOX was tested against Gram-positive and CIP against Gram negative species.

**Results:**

The most common organisms were SA (n=8,540), including 4,967 MSSA and 3,573 MRSA (58%), PSA (n=1,085), EC (n=840), and SP (n=781). Susceptibilities (%S) by year to DLX, LEV, and MOX are shown in the Table. For MRSA, %S to DLX increased from 81.0% to 87.0% over 6 years, while LEV declined from 36.5% to 34.7% and MOX from 36.8% to 34.7%. SP %S to all FQ was stable at 99.7–100%. EF %S to DLX increased from 64.6% to 80.3% with smaller increases for LEV (5%) and MOX (3%). EC %S to DLX increased from 59.4% to 74.4%, and also increased for LEV (66.7% to 75.2%) and CIP (61.8 to 76.0%). PSA %S to DLX increased from 64.9% to 73.7%, as did %S to LEV (69.5% to 74.7%). The PSA %S to CIP declined slightly from 82.5% to 79.8%.

**Conclusion:**

An increase in %S to FQs was observed for most ABSSSI pathogens, including EC, EF, and PSA. MRSA %S to DLX increased from 2017 to 2022, while %S to LEV and MOX declined. The decreased use of FQs may have led to improved susceptibility for DLX and as such it may be a good treatment option for ABSSSI.

**Disclosures:**

**Cecilia G. Carvalhaes, MD, PhD**, AbbVie: Grant/Research Support|bioMerieux: Grant/Research Support|Cipla: Grant/Research Support|CorMedix: Grant/Research Support|Melinta: Grant/Research Support|Pfizer: Grant/Research Support **Dee Shortridge, PhD**, Melinta: Grant/Research Support|Shionogi: Grant/Research Support **Leonard R. Duncan, PhD**, AbbVie: Grant/Research Support|Basilea: Grant/Research Support|CorMedix: Grant/Research Support|Melinta: Grant/Research Support|Pfizer: Grant/Research Support **Mike Huband, BS**, Melinta: Grant/Research Support **Mariana Castanheira, PhD**, AbbVie: Grant/Research Support|Basilea: Grant/Research Support|bioMerieux: Grant/Research Support|Cipla: Grant/Research Support|CorMedix: Grant/Research Support|Entasis: Grant/Research Support|Melinta: Grant/Research Support|Paratek: Grant/Research Support|Pfizer: Grant/Research Support|Shionogi: Grant/Research Support

